# Inhibition of the Mitotic Exit Network in Response to Damaged Telomeres

**DOI:** 10.1371/journal.pgen.1003859

**Published:** 2013-10-10

**Authors:** Mauricio Valerio-Santiago, Ana Isabel de los Santos-Velázquez, Fernando Monje-Casas

**Affiliations:** Centro Andaluz de Biología Molecular y Medicina Regenerativa/Departamento de Genética, Universidad de Sevilla, Sevilla, Spain; University of California San Francisco, United States of America

## Abstract

When chromosomal DNA is damaged, progression through the cell cycle is halted to provide the cells with time to repair the genetic material before it is distributed between the mother and daughter cells. In *Saccharomyces cerevisiae*, this cell cycle arrest occurs at the G2/M transition. However, it is also necessary to restrain exit from mitosis by maintaining Bfa1-Bub2, the inhibitor of the Mitotic Exit Network (MEN), in an active state. While the role of Bfa1 and Bub2 in the inhibition of mitotic exit when the spindle is not properly aligned and the spindle position checkpoint is activated has been extensively studied, the mechanism by which these proteins prevent MEN function after DNA damage is still unclear. Here, we propose that the inhibition of the MEN is specifically required when telomeres are damaged but it is not necessary to face all types of chromosomal DNA damage, which is in agreement with previous data in mammals suggesting the existence of a putative telomere-specific DNA damage response that inhibits mitotic exit. Furthermore, we demonstrate that the mechanism of MEN inhibition when telomeres are damaged relies on the Rad53-dependent inhibition of Bfa1 phosphorylation by the Polo-like kinase Cdc5, establishing a new key role of this kinase in regulating cell cycle progression.

## Author Summary

A key aspect of the division of cells is that the genomic material must be carefully duplicated, protected from damage, and correctly distributed during this process. When the cells detect problems that affect the integrity or the proper distribution of the genome, they trigger different surveillance mechanisms to stop the division process until the problem is fixed. The functionality of some of these surveillance mechanisms depends on the inhibition of the final stages of cell division, a process known as mitotic exit. This is the case for the DNA damage checkpoint (DDC), which is triggered by chromosomal DNA damage. Here, we propose that the inhibition of mitotic exit is specifically required by the DDC only when the telomeres (the chromosomal ends) are damaged. Additionally, we have demonstrated that the DDC blocks mitotic exit by inhibiting the key cell cycle regulator Cdc5, which triggers the inactivation of Bfa1 and Bub2, two negative regulators of the mitotic exit process.

## Introduction

During mitosis, different surveillance mechanisms ensure that the replicated genomic material is protected from damage and correctly distributed between the daughter and the mother cells. In this way, chromosomal DNA damage triggers a stress response pathway known as the DNA damage checkpoint (DDC) [1], which arrests the cell cycle to provide the cells with time to repair the genomic material before further progressing into mitosis. The cells must also ensure that all the chromosomes are attached to the spindle, a bipolar array of microtubules that allows for the segregation and distribution of the chromosomes between the daughter and mother cells. The proper attachment of all kinetochores to the spindle is monitored by the spindle assembly checkpoint (SAC), which otherwise delays the onset of anaphase [2]. Finally, in cells that display some type of asymmetry during mitosis, as it is the case of the budding yeast *Saccharomyces cerevisiae*, it is also essential that the spindle be correctly positioned with respect to the division site. In this organism, this is ensured by the spindle position checkpoint (SPOC), which restrains mitotic exit when the spindle is not properly aligned along the mother-bud axis and perpendicular to the bud neck [3]. A similar mechanism, the centrosome orientation checkpoint (COC), has also been described in *Drosophila* male germline stem cells [3]. The COC delays the commitment to mitosis in case of centrosome misorientation.

Despite the diverse signals that trigger the DDC, the SAC and the SPOC, as well as the different cell cycle stages where these surveillance mechanisms are triggered, the three checkpoints have been shown to inhibit mitotic exit in *S. cerevisiae* by activating the two-component GTPase-activating protein (GAP) Bfa1-Bub2 [4]. This GAP inhibits Tem1, a GTPase that initiates signaling by the Mitotic Exit Network (MEN) [5]. Activation of the MEN drives a sustained release of the Cdc14 phosphatase out of the nucleolus in anaphase, where it is sequestered from G1 to metaphase by its inhibitor Cfi1/Net1 [6], [7]. Once released into the nucleus and the cytoplasm, Cdc14 reverts the phosphorylation events triggered by the mitotic cyclin-CDK complexes, leading to their inactivation and the completion of mitosis [5]. During a normal cell cycle, the activity of Bfa1-Bub2 is regulated through phosphorylation. At the onset of anaphase, Bfa1 is phosphorylated by the Polo-like kinase Cdc5, which inactivates the GAP and promotes MEN signaling [4], [8], [9]. Cdc5 also plays an essential role during mitotic exit by recruiting the MEN kinase Cdc15 to the spindle pole bodies (SPBs, the yeast equivalent of the centrosomes) [10]. When the SAC or the SPOC are triggered, however, Bfa1 is maintained in a hypo-phosphorylated and therefore active state, which restrains mitotic exit [4]. The protein kinase Kin4 plays a key role in the SPOC by promoting the inhibitory action of Bfa1 on MEN signaling. When the spindle is not properly positioned, Kin4 phosphorylates Bfa1 impeding its inactivation by Cdc5 [11], [12]. In addition, Kin4 actively excludes Bfa1 from the SPBs after SPOC activation [13]. Since Tem1 localization to the SPBs depends on Bfa1 [14] and it is essential for MEN signaling [15], this also contributes to the inactivation of mitotic exit under these circumstances.

While the role of Bfa1 and Bub2 in the inhibition of mitotic exit after the activation of the SAC and the SPOC has been extensively studied, the mechanism by which these proteins prevent MEN function after the generation of DNA damage is still unclear. In *S. cerevisiae*, a central regulator of the DDC is the Mec1 kinase, the yeast homolog to mammalian ATR, which activates Chk1 and Rad53, two kinases that form parallel branches of the Mec1-dependent DDC pathway [1]. After DNA damage, Chk1 inhibits the metaphase to anaphase transition by stabilizing securin (Pds1) and therefore maintaining separase (Esp1) inactive [16]. Rad53 also inhibits the metaphase to anaphase transition by regulating Pds1 stability, but it has been additionally shown to prevent mitotic exit by regulating Bfa1-Bub2 [4], [17]. The mechanism by which Rad53 inhibits Bfa1-Bub2 is, at present, not clear. The kinase activity of Cdc5 was initially found to be high in DNA-damaged cells [18], while Bfa1 was described to be phosphorylated but active in a Rad53-dependent manner after DDC activation [4]. Thus, and since phosphorylation of Bfa1 by Cdc5 inhibits its activity, it was formally proposed that, in contrast to the SAC and the SPOC, the inhibition of mitotic exit after DNA damage is independent of the phosphorylation of Bfa1 by Cdc5 [4], and that, under these circumstances, Rad53 would be then controlling the activity of Bfa1 by a yet-unknown mechanism [4]. More recently, however, it has been demonstrated that Cdc5 is in fact partially inhibited by Rad53 after the generation of DNA damage [19]. This inhibition restrains the elongation of the spindle by preventing the inactivation of the anaphase-promoting complex cofactor Cdh1, thus limiting the accumulation of the bimC kinesin family proteins Cin8 and Kip1 [19]. Therefore, and based on these new observations, it is necessary to reevaluate whether Bfa1 phosphorylation by Cdc5 needs to be actively prevented also after DDC activation in order to maintain the GAP in an active state and prevent MEN signaling.

Here, we have analyzed the regulation of mitotic exit in response to DNA damage. We have determined the consequences of the lack of Bfa1 activity on the functionality of the DDC in response to a wide variety of chromosomal DNA damage. Based on our results, we propose that the inhibition of MEN signaling is essential when telomeres are damaged, but it is not a general requirement for the functionality of the DDC. Additionally, we have analyzed the mechanism by which the DDC impedes MEN signaling, and the role of Cdc5 in this process. We have demonstrated that the Rad53-dependent inhibition of Cdc5 after DNA damage is essential to maintain Bfa1 in a hypo-phosphorylated form that inactivates Tem1 and thus inhibits mitotic exit.

## Results

### Bfa1 plays a specific role in the response to damaged telomeres

**Figure 1:**
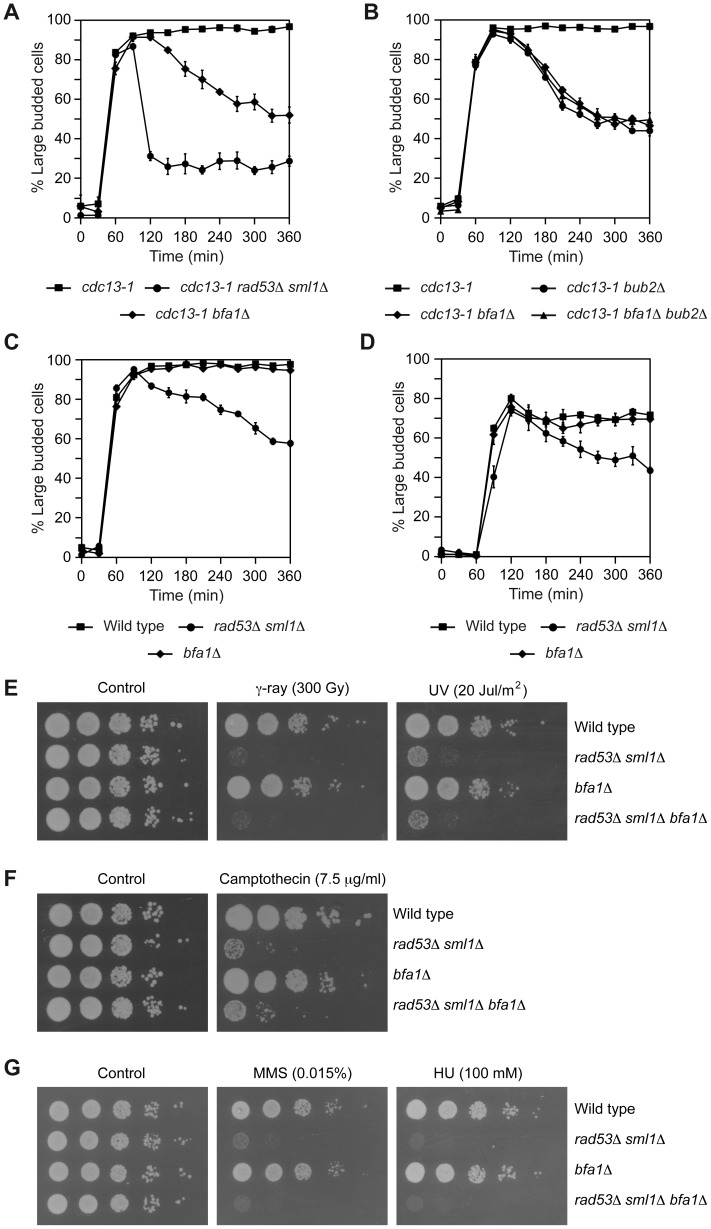
Inhibition of mitotic exit is specifically required after DNA damage to telomeres. (A–B) *cdc13-1* (F965), *cdc13-1 rad53*Δ *sml1*Δ (F1662), *cdc13-1 bfa1*Δ (F1023), *cdc13-1 bub2*Δ (F1787) and *cdc13-1 bfa1*Δ *bub2*Δ (F1786) cells were grown in YPD at 23°C and arrested in G1 with pheromone. Cells were then released into YPD at 34°C and the percentages of large budded cells were determined at the indicated time points. Error bars indicate SD (n = 3). (C) Wild type (F1587), *rad53*Δ *sml1*Δ (F1019) and *bfa1*Δ (F1589) cells were grown in YPD at 25°C and arrested in G1 with pheromone. After pheromone washout, cells were released into YPD containing zeocin (50 µg/µl) and the percentages of large budded cells were determined at the indicated time points. Error bars indicate SD (n = 3). (D) *rad53*Δ *sml1*Δ (F1679), *bfa1*Δ (F1606), and otherwise wild type cells (F1496) carrying an HO recognition site in chromosome II and the HO endonuclease under the control of a galactose-inducible promoter were grown at 25°C in rich media with 2% raffinose and synchronized in G1 using pheromone. After release from the G1-arrest, cells were grown at 25°C in rich media with 4% galactose to induce HO expression. The percentages of large budded cells were determined at the indicated time points. Error bars indicate SD (n = 3). (E–G) Wild type (F1587), *rad53*Δ *sml1*Δ (F1019), *bfa1*Δ (F1589) and *rad53*Δ *sml1*Δ *bfa1*Δ (F1661) cells were plated by spotting 10-fold serial dilutions of a liquid culture (OD_600_ = 0.3) on YPD (E–F) or minimal media (G) plates and then incubated at 30°C. (E) Before being plated, cells were irradiated with γ-rays (300 Gy) or UV (20 Jul/m^2^). (F–G) The cells were plated in media containing camptothecin (7.5 µg/µl), MMS (0.015%) or HU (100 mM), as indicated.

The role of Bfa1-Bub2 in the response to DNA damage was originally determined by using the *cdc13-1* allele [4], [20]. Cdc13 is an essential protein that protects the telomere from degradation and regulates telomerase activity [21]–[24]. Cells carrying the *cdc13-1* mutation cannot “cap” the telomeres and accumulate single-stranded DNA at the restrictive temperature, which triggers a DDC-dependent cell cycle arrest in G2/M [21], [25]. Accordingly, *cdc13-1* cells synchronized in G1 using pheromone accumulated as large budded cells with an undivided nucleus after their release into pheromone-free medium at 34°C, while DDC-deficient *cdc13-1 rad53*Δ *sml1*Δ cells could not hold this arrest (Figure 1A, [26]; deletion of *SML1* is necessary to bypass the lethality associated to the inhibition of the ribonucleotide reductase and the subsequent reduced dNTPs levels in *rad53*Δ cells [27]). Approximately 30% of *cdc13-1 rad53*Δ *sml1*Δ cells still remained arrested, but this percentage was reduced to only 15% when *CHK1* was also deleted and the other branch of the DDC was thus additionally inactivated (Figure S1A). The metaphase arrest observed for *cdc13-1* cells at the restrictive temperature was also dependent on both Bfa1 and Bub2 (Figures 1A and B, [20]), which indicates that inhibition of MEN by the two-component GAP must also be ensured after the telomeres are damaged.

In order to analyze whether inactivation of the MEN is a general requirement for the functionality of the DDC, we tested the capacity of *bfa1*Δ cells to maintain a DDC-dependent cell cycle arrest in the presence of other types of chromosomal damage. Wild type, *rad53*Δ *sml1*Δ, and *bfa1*Δ cells were synchronized in G1 using pheromone. After pheromone washout, cells were released into medium containing zeocin, a chemical that generates DNA double strand breaks (DSBs) [28]. The DNA damage generated by zeocin led to an accumulation of large budded cells in the wild type strain (Figures 1C and S1C), as previously observed for the *cdc13-1* mutant at the restrictive temperature. The cell cycle arrest induced by zeocin treatment was dependent on the functionality of the DDC, since it could not be held in *rad53*Δ *sml1*Δ cells (Figures 1C and S1C). However, and in contrast to what was observed for the *cdc13-1 bfa1*Δ mutant at 34°C, zeocin-treated *bfa1*Δ cells accumulated as large budded (Figures 1C and S1C). The specific requirement for Bfa1 after telomere damage cannot be attributed to different levels of checkpoint activation, since it was still observed in *cdc13-1* cells growing at 37°C and wild type cells treated with zeocin at the same temperature, for which the extent of DDC activation, as measured by the effect of *RAD53* deletion on the percentage of large budded cells, was similar (Figure S1D and E). Inhibition of MEN signaling was also not necessary to restrain cell cycle progression in response to the generation of a single unrepaired DSB induced by expression of the HO-endonuclease in cells in which an HO recognition site was introduced in chromosome II and that also carried a *MATa* allele that cannot be cleaved by HO (*MATa-inc*). The induction of the DSB in these cells led to a Rad53-dependent cell cycle arrest (Figures 1D and S1F). However, this DSB-induced arrest could still be maintained in *bfa1*Δ cells (Figures 1D and S1F). Therefore, our results indicate that, while necessary to block the cell cycle in response to uncapped telomeres, inhibition of mitotic exit is not essential for the DDC to maintain a cell cycle arrest after generation of DSBs in the DNA.

To further evaluate the role of Bfa1 in the response to DNA damage, we also assessed the survival of wild type, *rad53*Δ *sml1*Δ, and *bfa1*Δ cells after irradiation with gamma rays and UV, as well as their capacity to grow in the presence of different compounds that induce DNA damage (camptothecin, methyl methanesulfonate (MMS) and hydroxyurea (HU)). While, as expected, *rad53*Δ *sml1*Δ cells showed poor survival after irradiation with both gamma rays and UV (Figure 1E, [26]) and reduced viability in the presence of DNA-damaging chemicals due to an impairment of the DDC (Figures 1F and G, [29]), cells lacking Bfa1 behaved as wild type cells (Figures 1E, F and G). Therefore, our results suggest that rather than a general role in the protection against DNA damage, Bfa1 plays a more specific role in the response of the cell to the damage induced by a failure in telomere capping.

### Bfa1 hyper-phosphorylation is inhibited after DNA damage at telomeres

**Figure 2:**
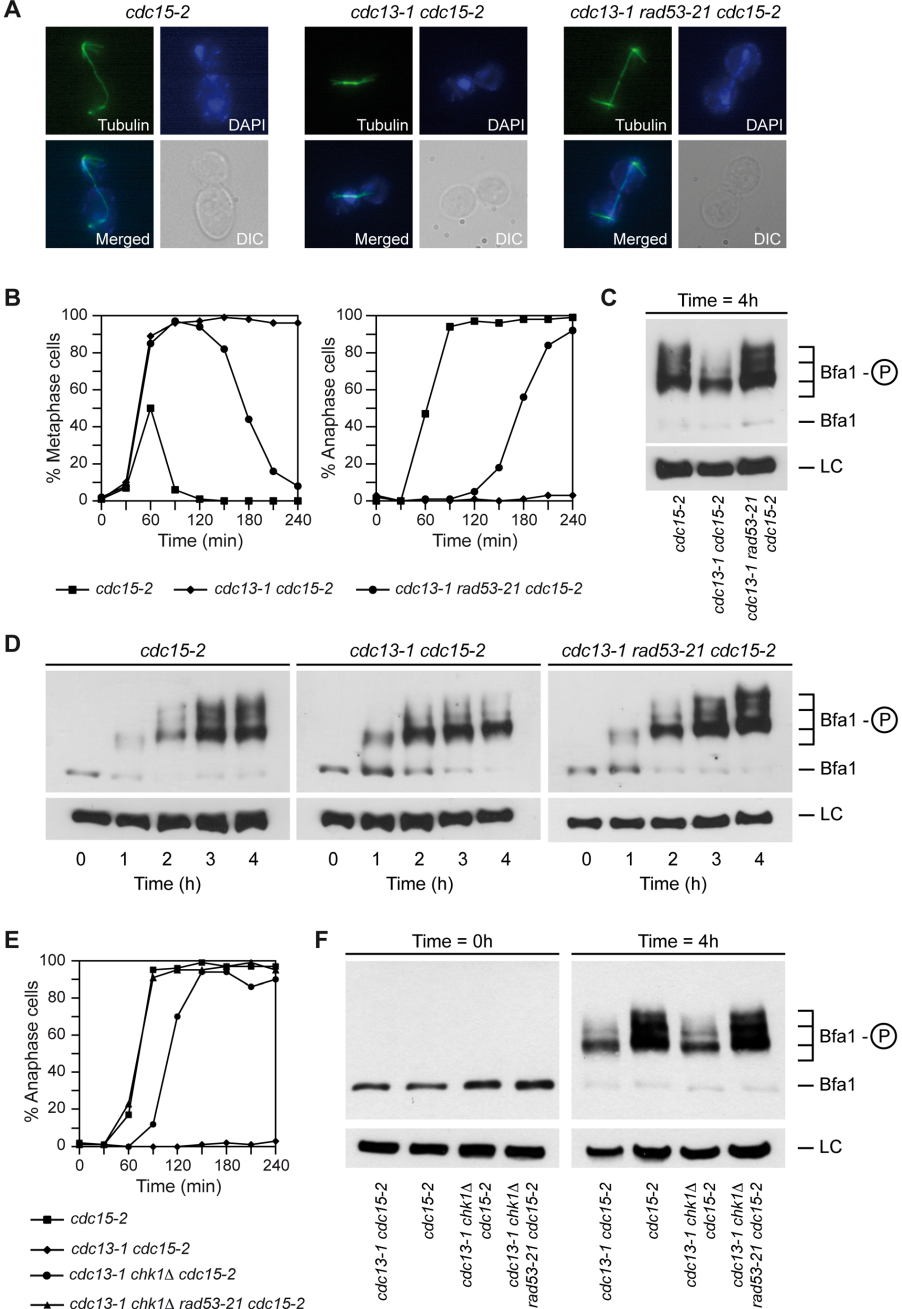
Bfa1 is hypo-phosphorylated by the DDC in a Rad53-dependent manner. (A–F) *cdc15-2* (F1492), *cdc13-1 cdc15-2* (F1488), *cdc13-1 rad53-21 cdc15-2* (F1453), *cdc13-1 chk1*Δ *cdc15-2* (F1782), and *cdc13-1 rad53-21 chk1*Δ *cdc15-2* (F1784) cells expressing 3HA-Bfa1 were arrested in G1 with pheromone in YPD at 23°C, and then released into pheromone-free medium at 34°C. (A) Representative images of the cells (DIC), as well as the spindle (tubulin) and nuclear (DAPI) morphologies, are displayed for each strain at the final cell cycle arrest. (B, E) The percentages of metaphase and/or anaphase cells were determined for each of the strains at the indicated time points. (C,D,F) 3HA-Bfa1 phosphorylation was analyzed by Western blot at the indicated time points. An unspecific band was used as a loading control (LC).

During a normal cell cycle, the activity of Bfa1-Bub2 is regulated through phosphorylation. At the onset of anaphase, Bfa1 is phosphorylated by Cdc5, which inactivates the GAP and allows for MEN signaling [4], [8]. When chromosomes are not correctly attached to the spindle and the SAC is activated, Bfa1 is maintained in a hypo-phosphorylated and therefore active state, which restrains mitotic exit [4]. The same happens when the spindle is not properly aligned and the spindle position checkpoint (SPOC) is activated [4]. In order to analyze the molecular mechanism by which damaged telomeres trigger a Bfa1-dependent inhibition of mitotic exit, we determined the phosphorylation status of a N-terminal 3HA-tagged version of Bfa1 in cells carrying the *cdc13-1* allele. We also analyzed the phosphorylation of Bfa1 in *cdc15-2* cells, which cannot exit mitosis at the restrictive temperature due to a block in MEN signaling downstream of Bfa1 [9], [30]. The cells were synchronized in G1 at the permissive temperature using pheromone, and then released into pheromone-free medium at the restrictive temperature. As expected, the *cdc15-2* mutant arrested in anaphase, while *cdc13-1 cdc15-2* cells were blocked already in metaphase due to the activation of the DDC (Figures 2A and B). This also indicates that the N-terminal 3HA-tag of Bfa1 does not affect its functionality. Phosphorylation of Bfa1 was analyzed in these cells after optimization of the experimental conditions to detect as many modified forms of the protein as possible. Bfa1 was heavily modified in the *cdc15-2* arrest (Figures 2C and D). These modifications were caused by phosphorylation of the protein, since they were no longer detected after phosphatase treatment (Figure S2A). Interestingly, activation of the DDC in *cdc13-1* cells at the restrictive temperature prevented hyper-phosphorylation of Bfa1 (Figures 2C and D). Although Bfa1 was still phosphorylated to some extent, the two highest phosphorylated forms of the protein disappeared in the *cdc13-1* arrest when compared to the *cdc15-2* mutant.

We next analyzed the role of Rad53 in the regulation of Bfa1 activity by determining the phosphorylation status of Bfa1 in *cdc13-1 rad53-21* cells. The checkpoint-deficient *rad53-21* allele does not need the additional deletion of *SML1* [31], which simplifies strain construction. As observed for *cdc13-1 rad53*Δ *sml1*Δ cells, the *cdc13-1 rad53-21* mutant could not hold the metaphase arrest at the restrictive temperature (Figure 2B). Accordingly, *rad53-21* cells also displayed low survival after irradiation with gamma rays and UV (Figure S2D) and reduced viability in media containing camptothecin, zeocin, MMS or HU (Figures S2E and F). In order to block anaphase progression, the *cdc13-1 rad53-21* mutant also carried the *cdc15-2* allele. Analysis of the spindle and nuclear morphologies indicated that the *cdc13-1 rad53-21 cdc15-2* cells indeed elongated the spindle and arrested at this cell cycle stage (Figures 2A and B). Bfa1 was found to be hyper-phosphorylated in *cdc13-1 rad53-21 cdc15-2* cells at the restrictive temperature to the same extent than in a *cdc15-2* mutant (Figures 2C and D). The results were similar when the *cdc14-3* allele was used instead of *cdc15-2* in order to promote the final anaphase arrest (unpublished observations). The inhibition of the hyper-phosphorylated forms of Bfa1 after telomere damage is dependent on Rad53 activity, and it is not exclusively associated to the *rad53-21* allele, since the phosphorylation pattern of the protein was the same in *cdc13-1 rad53*Δ *sml1*Δ *cdc15-2* cells at the restrictive temperature (Figures S2B and C). Interestingly, and even though Bfa1 was not necessary to hold cell cycle progression after zeocin treatment, the Rad53-dependent inhibition of Bfa1 phosphorylation was also observed when cells were treated with this compound (Figure S3A).

We also analyzed the role of other components of the DDC in the phosphorylation of Bfa1 when telomeres are damaged. Mec1 is the main sensor of the DNA damage response in *cdc13-1* cells, and triggers both the Rad53 and Chk1 branches of the DDC [1]. Accordingly, *cdc13-1 mec1*Δ *sml1*Δ cells did not arrest and Bfa1 was found to be hyper-phosphorylated at the non-permissive temperature (Figures S3B and C). On the contrary, Tel1 was not required to maintain the cell cycle block when *cdc13-1* cells were shifted at the restrictive temperature (Figure S3C), and the hyper-phosphorylation of Bfa1 was effectively inhibited in the *cdc13-1 tel1*Δ mutant (Figure S3B). This is in agreement with the fact that Rad53 is still phosphorylated and active in this mutant [32]. Finally, we checked whether the Chk1-dependent branch of the DDC plays any role in the regulation of Bfa1 phosphorylation. Although the *cdc13-1 chk1*Δ *cdc15-2* mutant was also unable to hold the metaphase arrest induced by damaged telomeres at the restrictive temperature (Figure 2E), Bfa1 was still hypo-phosphorylated in these cells in a Rad53-dependent manner (Figure 2F). Therefore, our results demonstrate that Rad53, but not Chk1, inhibits the hyper-phosphorylation of Bfa1 after DDC activation.

### Rad53 inhibits phosphorylation of Bfa1 by Cdc5

**Figure 3:**
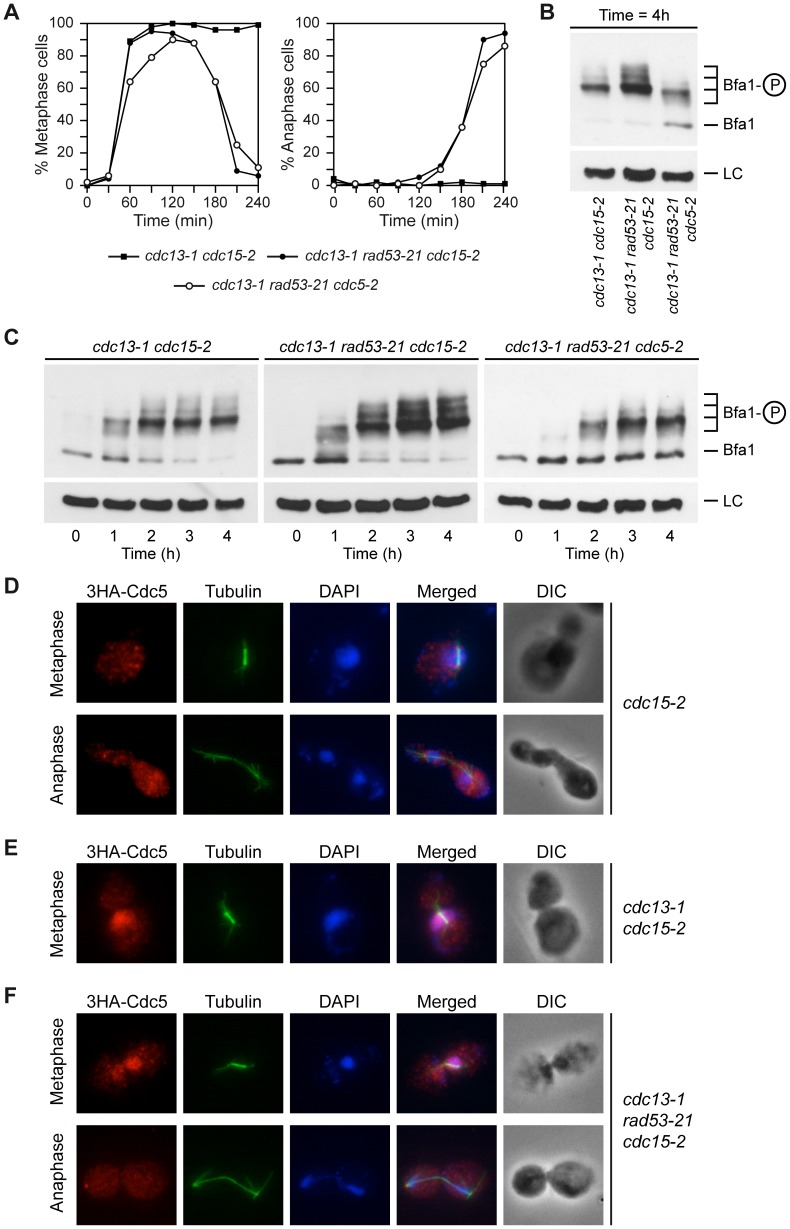
Rad53 prevents Bfa1 phosphorylation by the Polo-like kinase Cdc5. (A–C) *cdc13-1 cdc15-2* (F1488), *cdc13-1 rad53-21 cdc15-2* (F1453) and *cdc13-1 rad53-21 cdc5-2* (F1316) cells expressing 3HA-Bfa1 were arrested in G1 with pheromone in YPD at 23°C, and then released into pheromone-free medium at 34°C. (A) The percentages of metaphase and anaphase cells were determined for each of the strains at the indicated time points. (B–C) 3HA-Bfa1 phosphorylation was analyzed by Western blot at the indicated time points. An unspecific band was used as a loading control (LC). (D–F) 3HA-Cdc5 localization in *cdc15-2* (F1822), *cdc13-1 cdc15-2* (F1824), and *cdc13-1 rad53-21 cdc15-2* (F1825) cells. The cells were arrested in G1 at 23°C and then released at 34°C. The morphology of the cells (DIC), the spindle (tubulin) and nuclear (DAPI) morphologies, as well as a merged image (merged) are also displayed.

The Polo-like kinase Cdc5 phosphorylates Bfa1 in anaphase, which inhibits the GAP and activates MEN signaling [4]. It has been recently demonstrated that Rad53 partially inactivates Cdc5 after induction of DNA damage to protect Cdh1 from inhibition and therefore restrain spindle elongation [19]. Therefore, the partial inactivation of Cdc5 by Rad53 could also be determining the DDC-dependent hypo-phosphorylation of Bfa1. To test this hypothesis, the *cdc5-2* allele was introduced in *cdc13-1 rad53-21* cells. At the restrictive temperature, *cdc5-2* cells cannot exit mitosis. This phenotype can be recovered by deleting *BFA1*, which indicates that Cdc5-2 is specifically impaired in its ability to inactivate Bfa1 [4]. After their synchronization in G1 using pheromone, *cdc13-1 rad53-21 cdc5-2* cells were released at 34°C. Even though Cdc5-inactivation delayed cell cycle progression [4], [17], the cells finally reached anaphase (Figure 3A), as previously shown [17], [26]. However, and according to our hypothesis, the hyper-phosphorylation of Bfa1 observed in the *cdc13-1 rad53-21 cdc15-2* mutant was prevented in *cdc13-1 rad53-21 cdc5-2* cells (Figures 3B and C). We obtained the same results using the *cdc5-as1* allele to inactivate Polo-like kinase activity. Cdc5-as1 can be conditionally inhibited by adding the CMK-C1 ATP analog to the medium [33]. G1-synchronized *cdc13-1 rad53-21 cdc5-as1 cdc15-2* cells were allowed to enter the cell cycle at 34°C in pheromone-free medium containing or not the CMK-C1 inhibitor. As previously observed with the *cdc5-2* allele, inactivation of Cdc5-as1 prevented the hyper-phosphorylation of Bfa1 in the cells that escaped the DDC-dependent metaphase arrest (Figures S4A and B). The hyper-phosphorylated forms of Bfa1 were also absent in anaphase-arrested *cdc5-2* cells at the restrictive temperature (our unpublished observations, [11]). This suggests that the two most heavily phosphorylated forms of Bfa1 are indicative of the Cdc5-dependent inhibition of the GAP in anaphase.

Bfa1 localizes to the cytoplasmic side of the SPBs during mitosis [14], and its localization was not significantly affected in *cdc13-1* cells at the restrictive temperature (Figure S5G) or after treatment with zeocin (our unpublished observations). Cdc5 also localizes to the SPBs during mitosis, where it phosphorylates Bfa1 (Figure 3D, [5]). Interestingly, 3HA-Cdc5 strongly accumulated in the nucleus in *cdc13-1* cells at the non-permissive temperature (Figure 3E). This accumulation was also observed in *cdc13-1 rad53-21* cells while in metaphase. However, the cells that managed to escape the DDC-induced arrest released 3HA-Cdc5 from the nucleus and the protein could be observed on the SPBs during anaphase, as in wild type cells (Figure 3F). Although the strong accumulation of Cdc5 in the nucleus did not allow us to assess whether Polo kinase is loaded on the SPBs during the DDC-dependent arrest, our results suggest that Rad53 may additionally impair localization of Cdc5 to the SPBs, which would contribute to the inhibition of Bfa1 phosphorylation by Polo kinase.

### Bfa1 phosphorylation in telomere-damaged cells does not depend on Kin4 or CDK

**Figure 4:**
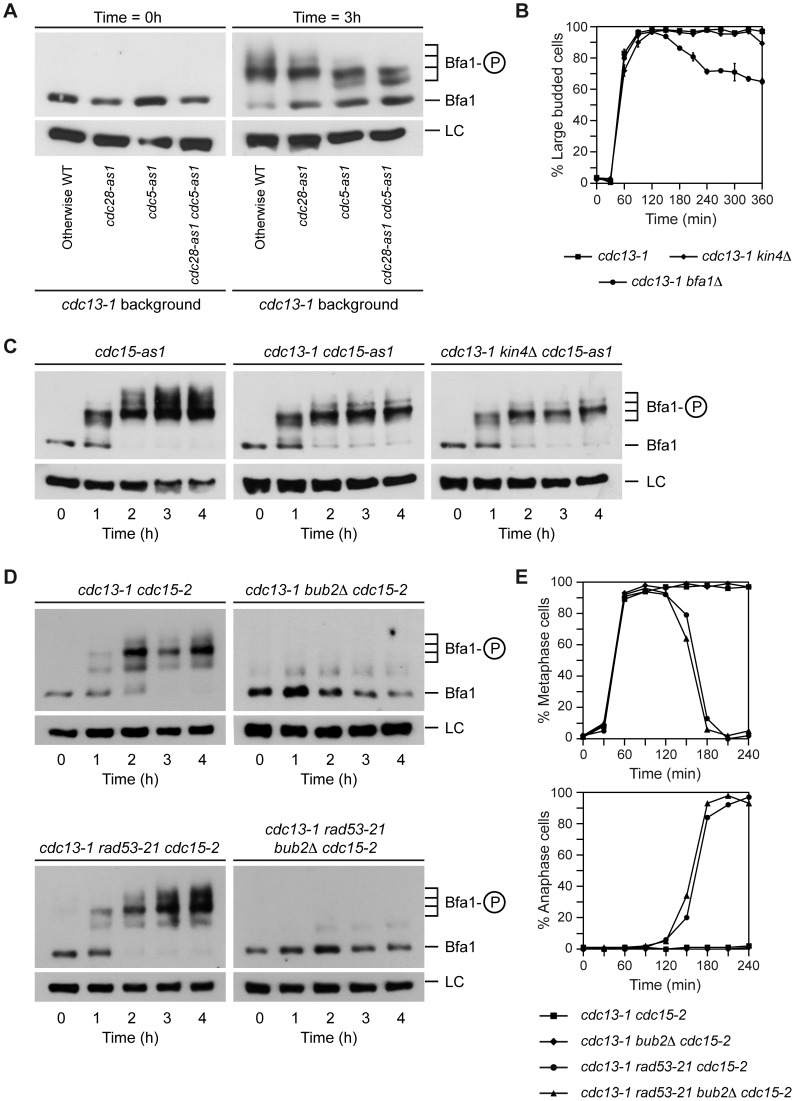
Bfa1 phosphorylation in the DDC-induced arrest is not dependent on CDK or Kin4. (A) Cells expressing 3HA-Bfa1 and carrying the *cdc13-1* allele alone (F1182) or in combination with either *cdc28-as1* (F1184), *cdc5-as1* (F1280) or both analogue-sensitive alleles (F1516) were arrested in G1 with pheromone in YPD at 23°C, released into pheromone-free medium containing the CMK-C1 (5 µM) and 1-NM-PP1 (500 nM) inhibitors, and incubated at 34°C. 3HA-Bfa1 phosphorylation was determined as in Figure 3C. (B) *cdc13-1* (F965), *cdc13-1 kin4*Δ (F973) and *cdc13-1 bfa1*Δ (F1023) cells were grown in YPD at 23°C, arrested in G1 with pheromone, and released into YPD at 34°C. The percentages of large budded cells were determined at the indicated time points. Error bars indicate SD (n = 3). (C) Cells expressing 3HA-Bfa1 and carrying the *cdc15-as1* allele alone (F1068) or in combination with *cdc13-1* (F1099) or *cdc13-1* and *kin4*Δ (F1117) were arrested in G1 with pheromone in YPD at 23°C, released into pheromone-free medium containing the 1-NA-PP1 (10 µM) inhibitor, and incubated at 34°C. 3HA-Bfa1 phosphorylation was determined as in Figure 3C. (D–E) *cdc13-1 cdc15-2* (F1488), *cdc13-1 bub2*Δ *cdc15-2* (F1879), *cdc13-1 rad53-21 cdc15-2* (F1453), and *cdc13-1 rad53-21 bub2*Δ *cdc15-2* (F1880) cells expressing 3HA-Bfa1 were arrested in G1 with pheromone in YPD at 23°C, and then released into pheromone-free medium at 34°C. (D) 3HA-Bfa1 phosphorylation was analyzed by Western blot at the indicated time points. An unspecific band was used as a loading control (LC). (E) The percentages of metaphase and anaphase cells were determined for each of the strains at the indicated time points.

Interestingly, Bfa1 was still phosphorylated to some extent after Cdc5 inactivation in *cdc13-1 rad53-21* cells, which indicates that this residual phosphorylation of the GAP is independent of the Polo-like kinase. Phosphorylation of a protein by Cdc5 is sometimes preceded by a priming phosphorylation of a Polo-binding site in the protein by cyclin-dependent kinases (CDKs) [34], [35]. However, Bfa1 phosphorylation in *cdc13-1* cells at the restrictive temperature is not dependent on CDK, since it was preserved after addition of the ATP analogue 1-NM-PP1 to *cdc13-1* cells carrying the analogue-sensitive *cdc28-as1* allele [36] (Figure 4A).

The protein kinase Kin4 plays a key role in promoting the inhibitory action of Bfa1 on MEN signaling after SPOC activation [11], [12]. Kin4 impedes the inhibition of Bfa1 by Cdc5 [12] and actively excludes the GAP from the SPBs after the SPOC is triggered [13]. Since Tem1 localization to this structure depends on Bfa1 and it is essential for MEN signaling [15], this exclusion also contributes to the inactivation of mitotic exit once the SPOC is activated. However, Kin4 does not determine Bfa1 phosphorylation in the *cdc13-1*-dependent arrest, and it is not necessary to maintain the functionality of the DDC (Figures 4B and C). Therefore, the kinase that phosphorylates Bfa1 under these conditions remains to be identified, as it is also yet unclear whether this phosphorylation plays a role in the functionality of the DDC. Interestingly, deletion of *BUB2* completely impairs the phosphorylation of Bfa1, including the Cdc5-independent phosphorylation observed in *cdc13-1* cells at the restrictive temperature (Figures 4D and 4E). This result suggests that the Cdc5-independent phosphorylation could take place at the SPBs, since Bub2 is necessary for Bfa1 to localize on this structure. In any case, and independently of the nature of this remnant phosphorylation, our results demonstrate that the Rad53-inhibition of Polo kinase activity not only restrains spindle elongation [19], but also promotes a Bfa1-dependent block of mitotic exit.

### Genetic analysis of the Rad53-Cdc5-Bfa1 branch of the DDC

**Figure 5:**
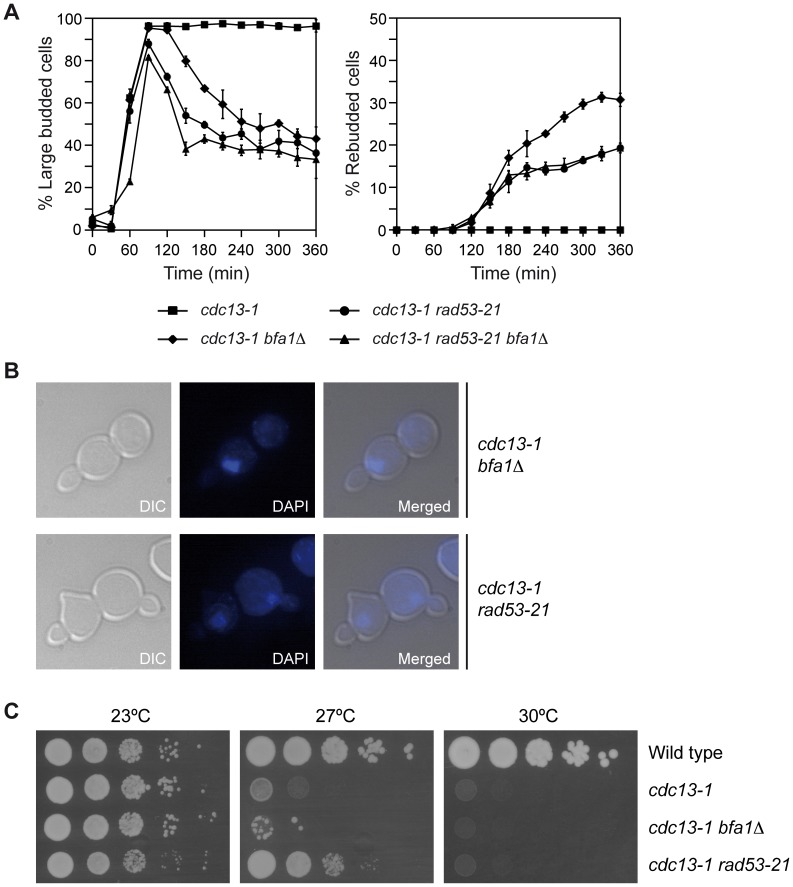
Mutants affected in Rad53 and Bfa1 display different phenotypes. (A–B) *cdc13-1* (F965), *cdc13-1 rad53-21* (F1228), *cdc13-1 bfa1*Δ (F1023) and *cdc13-1 rad53-21 bfa1*Δ (F1229) cells were grown in YPD at 23°C and arrested in G1 with pheromone. (A) Cells were then released into YPD at 34°C and the percentages of large budded and rebudded cells were determined at the indicated time points. Error bars indicate SD (n = 3). (B) Representative images of rebudded cells (DIC) and the nuclear morphology (DAPI) are also presented. (C) Wild type (F1587), *cdc13-1* (F965), *cdc13-1 rad53-21* (F1228) and *cdc13-1 bfa1*Δ (F1023) cells were plated by spotting 10-fold serial dilutions of a liquid culture (OD_600_ = 0.3) on YPD and then incubated at 23, 27 or 30°C.

According to our results, and even though mutants affected in either Rad53 or Bfa1 are deficient for the DDC in the presence of uncapped telomeres, they should display different phenotypes after induction of DNA damage. Rad53 should act upstream of Bfa1, and in its absence neither spindle elongation (Figure 2A) nor mitotic exit (Figure 1A) could be halted by the DDC. On the contrary, in a *bfa1*Δ mutant mitotic exit should occur without spindle elongation taking place. To test this, we analyzed cell cycle progression and budding in *cdc13-1 rad53-21* and *cdc13-1 bfa1*Δ cells. As previously indicated, and while the *cdc13-1* mutant at the restrictive temperature arrested as large budded cells, *cdc13-1 rad53-21* and *cdc13-1 bfa1*Δ cells could not hold the DDC-dependent arrest and entered a new cell cycle (Figure 5A). However, and as previously shown for *cdc13-1 rad53*Δ *sml1*Δ cells (Figure 1A), *cdc13-1 rad53-21* cells exited mitosis faster than *cdc13-1 bfa1*Δ, as evidenced by the faster decrease in large budded cells (Figure 5A). Furthermore, and as predicted, while most *cdc13-1 rad53-21* cells that escaped the arrest elongated their spindles, carried out cytokinesis, and accumulated as unbudded cells, *cdc13-1 bfa1*Δ cells did not elongate their spindles and most of them entered a new cell cycle without cytokinesis, which led to an accumulation of rebudded cells with a single nucleus (Figures 5A and 5B). A small percentage of *cdc13-1 rad53-21* cells also entered a new cell cycle without cytokinesis, but in this case, and in contrast to the *cdc13-1 bfa1*Δ mutant, some rebudded cells had elongated their spindles before exiting mitosis and therefore showed two separated nuclei (Figure 5B). The *cdc13-1 rad53-21 bfa1*Δ mutant behaved as the *cdc13-1 rad53-21* (Figure 5A), which further indicates that Bfa1 acts in the same pathway as Rad53. According to this, and also in agreement with our analysis of Bfa1 phosphorylation (Figure 2F), deletion of *BFA1* accelerated the mitotic exit phenotype of *cdc13-1 chk1*Δ cells (Figure S1B), which indicates that they are acting in different branches of the DDC.

The previous genetic analysis was consistent with the viability observed for *cdc13-1, cdc13-1 rad53-21*, and *cdc13-1 bfa1*Δ cells when compared to wild type cells in a drop test. None of the strains carrying the *cdc13-1* allele could grow already at 30°C (Figure 5C). At 27°C, *cdc13-1* showed very limited growth due to the cell cycle block induced by the presence of uncapped telomeres (Figure 5C). Interestingly, and even though their viability was reduced when compared to wild type cells, inactivation of Rad53 by the introduction of the checkpoint-deficient allele allowed *cdc13-1 rad53-21* cells to grow at this temperature to a higher extent than the *cdc13-1* mutant (Figure 5C). This indicates that the telomere damage induced by the *cdc13-1* allele at 27°C, despite leading to a strong DDC-dependent cell cycle arrest, does not severely affect viability of the cells when the checkpoint is not functional. It is also worth noting that the *cdc13-1 rad53-21* mutant could grow at 27°C because most of the cells that escaped the arrest elongated their spindles and carried out cytokinesis as they exited mitosis (Figure 5A). In contrast, we have demonstrated that *cdc13-1 bfa1*Δ cells mainly exited mitosis as mono-nucleated and rebudded cells, which are not viable (Figures 5A and B). Accordingly, the *cdc13-1 bfa1*Δ mutant at 27°C showed extremely limited viability at 27°C (Figure 5C). Together, our results are consistent with a dual role of the Rad53-dependent branch of the DDC, which not only prevents spindle elongation [19], but also impedes MEN signaling by maintaining Bfa1 in a hypo-phosphorylated and active form that inhibits Tem1.

**Figure 6:**
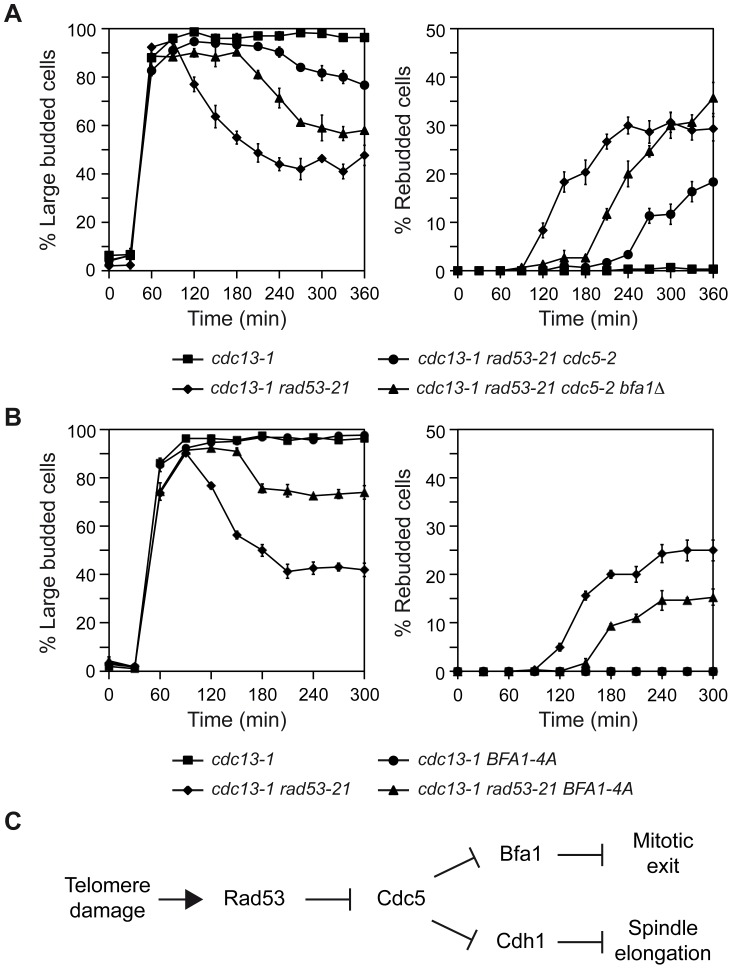
Genetic analysis of the Rad53-Cdc5-Bfa1 pathway. (A–B) *cdc13-1* (F965), *cdc13-1 rad53-21* (F1228), *cdc13-1 rad53-21 cdc5-2* (F1315), *cdc13-1 rad53-21 cdc5-2 bfa1*Δ (F1378), *cdc13-1 BFA1-4A* (F1396) and *cdc13-1 rad53-21 BFA1-4A* (F1397) cells were grown in YPD at 23°C and arrested in G1 with pheromone. Cells were then released into YPD at 34°C and the percentages of large budded and rebudded cells were determined at the indicated time points. Error bars indicate SD (n = 3). (C) Model for Rad53-dependent cell cycle arrest in response to damaged telomeres.

To further demonstrate that inhibition of Bfa1 phosphorylation by Cdc5 is essential for the Rad53-dependent cell cycle arrest originated after DNA damage, we analyzed the effect of mutations that affect Bfa1 phosphorylation on the mitotic exit phenotype of *rad53-21* cells. We first made use of the *cdc5-2* mutant, which can elongate the spindle and reach anaphase, but it is specifically impaired in its ability to phosphorylate and inactivate Bfa1 (Figures 3B and C, [4]). A *cdc13-1 rad53-21 cdc5-2* mutant at the restrictive temperature accumulated large budded cells due to the anaphase block (Figures 3A and 6A), although a small percentage of cells finally managed to escape the arrest (Figure 6A). According to our results, and if Cdc5-dependent phosphorylation of Bfa1 plays a key role in restraining mitotic exit after DDC activation, the arrest observed for *cdc13-1 rad53-21 cdc5-2* cells could be explained by a Bfa1-dependent inhibition of mitotic exit due to the inability of Cdc5 to phosphorylate the GAP, even though Rad53 is inactive. If so, we reasoned that deletion of *BFA1* should accelerate the mitotic exit phenotype of the *cdc13-1 rad53-21 cdc5-2* cells. Indeed, this was the case. Both the decrease in the percentage of large budded cells and the accumulation of rebudded cells were accelerated in the *cdc13-1 rad53-21 cdc5-2* mutant in the absence of *BFA1* (Figure 6A). Additionally, the percentage of rebudded cells was increased and matched the level observed in *cdc13-1 rad53-21* (Figure 6A). Exit from mitosis was still delayed in *cdc13-1 rad53-21 cdc5-2 bfa1*Δ cells as compared to the *cdc13-1 rad53-21* mutant probably due to problems in the progression through the cell cycle associated to the defect in Cdc5 [4], [17].

To strengthen our conclusions, we followed a parallel approach. The Bfa1-4A mutant cannot be efficiently phosphorylated by Cdc5 (Figures S5A and B, [37]). This mutant has been shown to symmetrically localize in anaphase [37], and its localization was not significantly affected after telomere damage (Figure S5G). Additionally, this mutant delayed mitotic exit in otherwise wild type cells [37] and it could hold the DDC-dependent cell cycle arrest induced by telomere damage (Figure 6B), zeocin treatment (Figure S5C) or the treatment with other DNA damaging agents (Figures S5D, E, and F), as expected by the fact that Cdc5-phosphorylation leads to inactivation of Bfa1 and promotes mitotic exit [37]. However, and according to our hypothesis, the Bfa1-4A mutant should delay the mitotic exit phenotype of *cdc13-1 rad53-21* cells, since even though Cdc5 would be active, it could not promote MEN signaling by the inhibition of Tem1's GAP. Indeed, *cdc13-1 rad53-21 BFA1-4A* cells exited mitosis at the restrictive temperature later than a *cdc13-1 rad53-21* mutant, as demonstrated by the decrease in the percentage of large-budded cells (Figure 6B). Additionally, the rebudding percentage was lower than in the case of *cdc13-1 rad53-21* cells (Figure 6B). MEN signaling was only delayed and not completely avoided probably due to the fact that an active Cdc5 kinase promotes mitotic exit not only through Bfa1 inactivation but also downstream of the GAP 9,10,38–40. The striking similarity between the *cdc13-1 rad53-21 BFA1-4A* and the *cdc13-1 rad53-21 cdc5-2* mutants demonstrates that the inhibition of Bfa1 phosphorylation by Polo-like kinase plays an essential role in the Rad53-dependent arrest generated by telomere damage.

## Discussion

The inhibition of MEN signaling by the SPOC is essential for anaphase cells with mispositioned spindles in order to provide them with time to reposition their spindles before exiting mitosis [3]. Interestingly, the functionality of mitotic checkpoints that are triggered earlier in the cell cycle is also dependent on the active inhibition of mitotic exit. In all the previous cases, this inhibition of MEN signaling is achieved by means of the activation of Bfa1/Bub2, a two-component GAP that negatively regulates Tem1 [4], [41]. While the SAC and the SPOC maintain Bfa1 in an active state by preventing its phosphorylation by the Polo-like kinase Cdc5, a different mechanism was originally proposed for the inhibition of MEN signaling by the DDC that would rely on a Rad53-dependent yet Cdc5-independent activation of Bfa1 [4], [17].

Here, we have analyzed the inhibition of mitotic exit after generation of chromosomal DNA damage and the resulting activation of the DDC. This inhibition of MEN signaling seems to be specifically required to face certain types of damage. In this way, and while Bfa1-Bub2 is necessary to maintain a DDC-dependent cell cycle arrest due to the generation of uncapped telomeres, the viability of a *bfa1*Δ mutant is not affected as compared with that of wild type cells after the treatment with a wide variety of DNA damaging agents, including UV or gamma-rays and compounds that generate DSBs or replicative stress. In agreement with this, *bub2*Δ cells do not show increased sensitivity to MMS or UV irradiation [42]. Even though uncapped telomeres resemble one half of a DSB, the checkpoint response partially differs for both structures [43]. Our results are extremely interesting, and provide a new piece of evidence that demonstrates that the cells respond differently to DNA DSBs than to the presence of uncapped telomeres. A possible explanation for these observations is that telomere damage could be triggering a weaker G2/M arrest that would rely on the additional inhibition of mitotic exit by Rad53. Our data, however, exclude this possibility, since Bfa1 is essential for the functionality of the DDC even when the DNA damage caused by telomere damage is increased to the same extent than in cells treated with zeocin. Instead, we favor a distinct alternative scenario, in which Bfa1-Bub2 could collaborate with the DDC to specifically protect the cells when telomere integrity is compromised. Interestingly, it has been recently shown that in cells from the marsupial *Potorous tridactylis*, laser-induced damage at the telomeres during anaphase causes cell cycle delay and cytokinesis failure [44]. Therefore, our results are in agreement with the idea of a specific requirement for mitotic exit inhibition when telomeres are damaged [44].

Our study also sheds light into the mechanism by which mitotic exit is inhibited in the presence of uncapped telomeres. To this end, we have analyzed the pattern of phosphorylation of Bfa1 both during a normal cell cycle and after checkpoint activation. Bfa1 is gradually phosphorylated throughout the cell cycle, reaching its highest phosphorylated status once the cells are in anaphase. The hyper-phosphorylation of Bfa1 during anaphase is dependent on the Polo-like kinase Cdc5, and it is therefore likely to represent the phosphorylation events that inactivate the Bfa1-Bub2 GAP at this cell cycle stage [8]. We have demonstrated that in response to DNA damage, Bfa1 is maintained in a hypo-phosphorylated and active state that inhibits Tem1 activity and therefore impedes mitotic exit. Additionally, we have also shown that the maintenance of the active form of Bfa1 after DDC activation relies on the Rad53-dependent inhibition of the Polo-like kinase Cdc5, but not on the Chk1-dependent branch of the checkpoint. Our results contrast with previous observations suggesting that Bfa1 was hyper-phosphorylated in a Rad53-dependent manner after DDC activation [4]. We do not know the basis for this discrepancy, which might be due to the use of a different genetic background or to the possibility that a 3HA-tag in the C-terminus [4] instead of the N-terminus of Bfa1 could be affecting its functionality after DDC activation. In any case, our results are fully consistent with our genetic analysis of the Rad53-Cdc5-Bfa1 pathway and are in agreement with a more recent report that demonstrates that Cdc5 activity is inhibited in a Rad53-dependent manner after DNA damage [19]. It is worth noting that the inhibition of the Cdc5-dependent hyper-phosphorylation of Bfa1 also takes place when cells are treated with zeocin, a compound that generates DSBs in the DNA. However, and as previously stated, our data clearly demonstrates that inhibition of Bfa1 is not required to maintain a DDC-dependent cell cycle arrest when DNA DSBs are generated. The specific requirement for mitotic exit inhibition after telomere damage suggests that additional mechanisms to inhibit cell cycle progression must be triggered when cells are exposed to other types of DNA damage that are not induced in the presence of uncapped telomeres, therefore relieving the requirement for the inactivation of the MEN by Bfa1-Bub2.

Ours and previous results demonstrate that Rad53 fulfills a dual role in preventing cell cycle progression in response to telomere damage: it restrains the metaphase-to-anaphase transition avoiding spindle elongation [19], but it also prevents mitotic exit by maintaining Bfa1 in an active state that blocks MEN signaling (Figure 6C). This dual action of Rad53 is in agreement with the different behavior in terms of mitotic exit observed at the restrictive temperature for *cdc13-1 rad53-21* cells, which can promote both spindle elongation and mitotic exit, and mutants in which exclusively either spindle elongation (*e.g.*, *cdc13-1 bfa1*Δ) or mitotic exit (*e.g.*, *cdc13-1 rad53-21 BFA1-4A*) is blocked. The inactivation of only one of the two Rad53-dependent functions in a *cdc13-1* background reduces the ability of the cells to escape from the G2/M arrest induced at the restrictive temperature as compared to *cdc13-1 rad53-21* cells. Therefore, the Cdc5-dependent inhibitory phosphorylation of Bfa1 by Rad53 plays a key role in the maintenance of the DDC-dependent cell cycle arrest determined by telomere damage. Interestingly, and besides regulating the activity of Cdc5, our results suggest that Rad53 may also regulate the localization of the Polo-like kinase to the outer plaque of the SPBs, where it phosphorylates and inhibits Bfa1 [4]. This could represent an additional mechanism by which Rad53 blocks mitotic exit in the presence of damaged telomeres.

Even though Rad53 inhibits the hyper-phosphorylation of Bfa1 by Cdc5, Bfa1 still displays a considerable degree of phosphorylation in the G2/M cell cycle arrest induced by the DDC in response to telomere damage. This phosphorylation of Bfa1 is Cdc5-independent, which is in agreement with previous observations [11], [41]. We have demonstrated that this remnant phosphorylation is also not dependent on Clb-CDK activity or the Kin4 kinase. Therefore, our results suggest the existence of an additional kinase that phosphorylates Bfa1 during metaphase. Since deletion of *BUB2* completely abrogates Bfa1 phosphorylation and the localization of Bfa1 and Bub2 on the spindle poles is interdependent [14], it is likely that this Cdc5-independent phosphorylation of Bfa1 could also take place at the SPBs. At present, however, the identity of this kinase remains to be established, as it is also not known what is the actual role of this phosphorylation in the regulation of the activity of Bfa1 during a normal cell cycle or in the functionality of the different cell cycle checkpoints. One possibility is that this phosphorylation could be involved in the regulation of Bfa1 loading onto the SPBs, since Bfa1 and Nud1 (its anchor on the SPB) preferentially co-immunoprecipitate in their phosphorylated forms [45].

Based on our results, the inhibition of Bfa1 phosphorylation by Cdc5 is a common theme for all the mitotic checkpoints that rely on the inhibition of mitotic exit. In agreement with this observation, overexpression of Cdc5 not only inhibits Bfa1-Bub2 activity in anaphase, but it is also able to bypass the cell cycle arrest induced by the activation of the DDC and the SAC [4], [40], [46]. These surveillance mechanisms therefore mainly diverge in the strategies by which the inhibition of Polo-kinase is achieved in each case. In this way, while Kin4 plays a critical role in the functionality of the SPOC [11], [12], this kinase is dispensable for the DDC and the SAC. Even though DDC activation in mammalian cells mainly blocks mitotic entry, exit from mitosis and cytokinesis are also restrained in these cells in response to DNA damage [44], [47], [48]. Furthermore, Polo-like Kinase I (Plk1) is inhibited after DNA damage in an ATM and ATR-dependent manner [49], [50], and it has been demonstrated to interact and co-localize in centrosomes and the midbody during mitosis with Chk2, the mammalian homolog of Rad53 [51]. Therefore, our data could provide new insights into common mechanisms by which exit from mitosis is prevented when the DNA is damaged in higher eukaryotes.

## Materials and Methods

### Strains

All strains are derivatives of W303 and are described in Table S1. Unless otherwise indicated, all the strains are *RAD5*. Strain F1333, which expresses Bfa1-GFP, was constructed by first linearizing the pRS304-*BFA1-GFP* with the NruI endonuclease and then integrating it within the *BFA1* promoter in F533, a strain in which the endogenous *BFA1* gene is deleted. Strain F1367, which expresses Bfa1-4A-GFP, was constructed as F1333, but integrating the pRS304-*BFA1-4A* plasmid [37].

### Immunolocalization and fluorescence microscopy

Immunofluorescence was performed as described in [15]. In brief, cells were fixed overnight at 4°C in 3.7% formaldehyde, washed twice with 0.1 M potassium phosphate buffer (pH 6.4), and resuspended in 1.2 M sorbitol/0.12 M KH_2_HPO_4_/0.033 M citric acid (pH 5.9). Fixed cells were digested for 15 min at 30°C with 0.1 mg/ml zymolyase-100T (US Biological) and 1/10 volume of glusulase (Perkin Elmer). Anti-tubulin (Abcam) and anti–rat FITC (Jackson ImmunoResearch) antibodies were used at 1∶200. 3HA-Cdc5 was detected using anti-HA antibody (HA.11; Covance) at 1∶500 and anti–mouse Cy3 antibody (Jackson ImmunoResearch Laboratories, Inc.) at 1∶1000. Samples for GFP and DAPI imaging were prepared as described in [15]. Microscope preparations were analyzed and imaged at 25°C using a DM6000 microscope (Leica) equipped with a 100×/1.40 NA oil immersion objective lens, A4, L5, and TX2 filters, and a DF350 digital charge-coupled device camera (Leica). Pictures were processed with LAS AF (Leica) and ImageJ (http://rsbweb.nih.gov/ij/) software.

### FACS analysis

Cells were fixed in 70% ethanol, incubated for 12 h in phosphate-buffered saline with 1 mg/ml of RNase A, and stained for 1 h with 5 µg/ml propidium iodide. After sonication of the sample to separate single cells, DNA content was analyzed in a FACSCalibur flow cytometer (Becton Dickinson).

### Western blot analysis

Protein extracts were prepared using the TCA precipitation method described in [11] and were loaded on 6% polyacrylamide gels. Electrophoresis was carried out using a SE600 Hoefer electrophoresis system. Samples subjected to phosphatase treatment were incubated with 150 U of bovine phosphatase alkaline (Sigma) for 12 h at 37°C in 50 mM Tris-HCl, 1 mM MgCl_2_ buffer (pH = 9). 3HA-Bfa1 was examined with monoclonal HA.11 (Covance) at 1∶5000 and anti–mouse HRP-linked antibodies (GE Healthcare) at 1∶10000. GFP-tagged proteins were analyzed using JL-8 Living colors® monoclonal antibody (Clontech) at 1∶1000 and anti-mouse HRP-linked antibody (GE Healthcare) at 1∶2000. Pgk1 levels were measured using anti-Pgk1 antibody (Invitrogen) at 1∶10000 and anti-mouse HRP-linked antibody (GE Healthcare) at 1∶20000. In all cases, the protein signal was detected using the Western Bright ECL system (Advansta).

## Supporting Information

Figure S1 Inhibition of mitotic exit is specifically required after DNA damage to telomeres. (A–B) *cdc13-1* (F965), *cdc13-1 rad53*Δ *sml1*Δ (F1662), *cdc13-1 chk1*Δ (F1238), *cdc13-1 rad53*Δ *sml1*Δ *chk1*Δ (F1830), *cdc13-1 bfa1*Δ (F1023), and *cdc13-1 chk1*Δ *bfa1*Δ (F1240) cells were grown in YPD at 23°C and arrested in G1 with pheromone. Cells were then released into YPD at 34°C and the percentages of large budded cells were determined at the indicated time points. Error bars indicate SD (n = 3).(C) FACS analysis corresponding to the experiment shown in Figure 1C. (D) *cdc13-1* (F965), *cdc13-1 rad53*Δ *sml1*Δ (F1662) and *cdc13-1 bfa1*Δ (F1023) cells were grown in YPD at 23°C and arrested in G1 with pheromone. Cells were then released into YPD at 37°C and the percentages of large budded cells were determined at the indicated time points. Error bars indicate SD (n = 3). (E) Wild type (F1587), *rad53*Δ *sml1*Δ (F1019) and *bfa1*Δ (F1589) cells were grown in YPD at 25°C and arrested in G1 with pheromone. Cells were then released into YPD containing zeocin (50 µg/µl) at 37°C and the percentages of large budded cells were determined at the indicated time points. Error bars indicate SD (n = 3). (F) FACS analysis corresponding to the experiment shown in Figure 1D.
(TIF)

Figure S2 Bfa1 hypo-phosphorylation after telomere damage depends on Rad53. (A) *cdc15-2* (F1492) and *cdc13-1 cdc15-2* (F1488) cells expressing 3HA-Bfa1 were arrested in G1 with pheromone in YPD at 23°C and released into pheromone-free medium at 34°C for 4 h. Protein extracts were treated (+PA) or not (−PA) with phosphatase and 3HA-Bfa1 phosphorylation was determined by Western blot. (B–C) *cdc15-2* (F1492), *cdc13-1 cdc15-2* (F1488) and *cdc13-1 rad53*Δ *sml1*Δ *cdc15-2* (F1684) cells expressing 3HA-Bfa1 were arrested in G1 with pheromone in YPD at 23°C, and then released into pheromone-free medium at 34°C. (B) The percentages of metaphase and anaphase cells were determined for each of the strains at the indicated time points. (C) 3HA-Bfa1 phosphorylation was analyzed by Western blot at the indicated time points. An unspecific band was used as a loading control (LC). (D–F) Wild type (F1587), *rad53-21* (F1591), *bfa1*Δ (F1589) and *rad53-21 bfa1*Δ (F1593) cells were plated by spotting 10-fold serial dilutions of a liquid culture (OD_600_ = 0.3) on YPD (D–E) or minimal media (F) plates and then incubated at 30°C. (D) Before being plated, cells were irradiated with γ-rays (300 Gy) or UV (20 Jul/m^2^). (E–F) The cells were plated in media containing camptothecin (7.5 µg/µl), zeocin (5 µg/µl), MMS (0.015%) or HU (100 mM), as indicated.
(TIF)

Figure S3 Inhibition of the hyper-phosphorylation of Bfa1 by the DDC. (A) *cdc15-2* (F1492) and *rad53*Δ *sml1*Δ *cdc15-2* (F1816) cells expressing 3HA-Bfa1 were arrested in G1 with pheromone in YPD at 23°C and released into pheromone-free medium at 34°C containing (+Zeocin) or not (−Zeocin) the DSB-generating compound zeocin (50 µg/ml). 3HA-Bfa1 phosphorylation was analyzed by Western blot at the indicated time points. An unspecific band was used as a loading control (LC). (B–C) *cdc15-as1* (F1068), *cdc13-1 cdc15-as1* (F1099), *cdc13-1 mec1*Δ *sml1*Δ *cdc15-as1* (F1620) and *cdc13-1 tel1*Δ *cdc15-as1* (F1619) cells expressing 3HA-Bfa1 were arrested in G1 with pheromone in YPD at 23°C, and then released at 34°C into pheromone-free medium containing the 1-NA-PP1 inhibitor (10 µM). (B) 3HA-Bfa1 phosphorylation was analyzed by Western blot at the indicated time points. An unspecific band was used as a loading control (LC). (C) The percentages of metaphase and anaphase cells were determined for each of the strains at the indicated time points.
(TIF)

Figure S4 Rad53 prevents the phosphorylation of Bfa1 by Cdc5. (A–B) *cdc13-1 cdc15-2* (F1488) and *cdc13-1 rad53-21 cdc5-as1 cdc15-2* (F1454) cells expressing 3HA-Bfa1 were arrested in G1 with pheromone in YPD at 23°C, released into pheromone-free medium containing or not (+DMSO) the CMK-C1 inhibitor (5 µM), and incubated at 34°C. (A) The percentages of metaphase and anaphase cells were determined for each of the strains at the indicated time points. (B) 3HA-Bfa1 phosphorylation was analyzed by Western blot at the indicated time points. An unspecific band was used as a loading control (LC).
(TIF)

Figure S5 Bfa1-4A is not hyper-phosphorylated by Cdc5 and is checkpoint-proficient. (A–B) Cells expressing Bfa1-GFP (F1333) or Bfa1-4A-GFP (F1367) were arrested in G1 with pheromone in YPD at 25°C, and released into pheromone-free medium at the same temperature. Phosphorylation was analyzed by Western blot at the indicated time points with an antibody that recognizes the GFP tag (α-GFP). Pgk1 was used as a loading control (α-PGK1). (C) Wild type (F1587), *rad53*Δ *sml1*Δ (F1019) and cells expressing *BFA1-4A* (F1367) were grown in YPD at 25°C and arrested in G1 with pheromone. Cells were then released at the same temperature into YPD containing zeocin (50 µg/µl), and the percentages of large budded cells were determined at the indicated time points. Error bars indicate SD (n = 3). (D–F) Wild type (F1587), *rad53-21* (F1591), *BFA1-4A* (F1367) and *rad53-21 BFA1-4A* (F1827) cells were plated by spotting 10-fold serial dilutions of a liquid culture (OD_600_ = 0.3) on YPD (D–E) or minimal media (F) plates and then incubated at 30°C. (D) Before being plated, cells were irradiated with γ-rays (300 Gy) or UV (20 Jul/m^2^). (E–F) The cells were plated in media containing camptothecin (7.5 µg/µl) or HU (100 mM), as indicated. (G) Analysis of Bfa1-GFP and Bfa1-4A-GFP localization to the SPBs in metaphase cells from strains F1902, F1903, F1826 and F1396 after release in YPD at 34°C from a previous G1-arrest.
(TIF)

Table S1 Strains. All strains used in this study are derivatives of W303. Unless otherwise indicated, all the strains are *RAD5*.
(PDF)
