# Supplementary figures and images for: Inhibition of the Mitotic Exit Network in Response to Damaged Telomeres
Source: PLoS Genet. 2013 Oct 10;9(10):e1003859. doi: 10.1371/journal.pgen.1003859 (PMC3794921; doi:10.1371/journal.pgen.1003859)

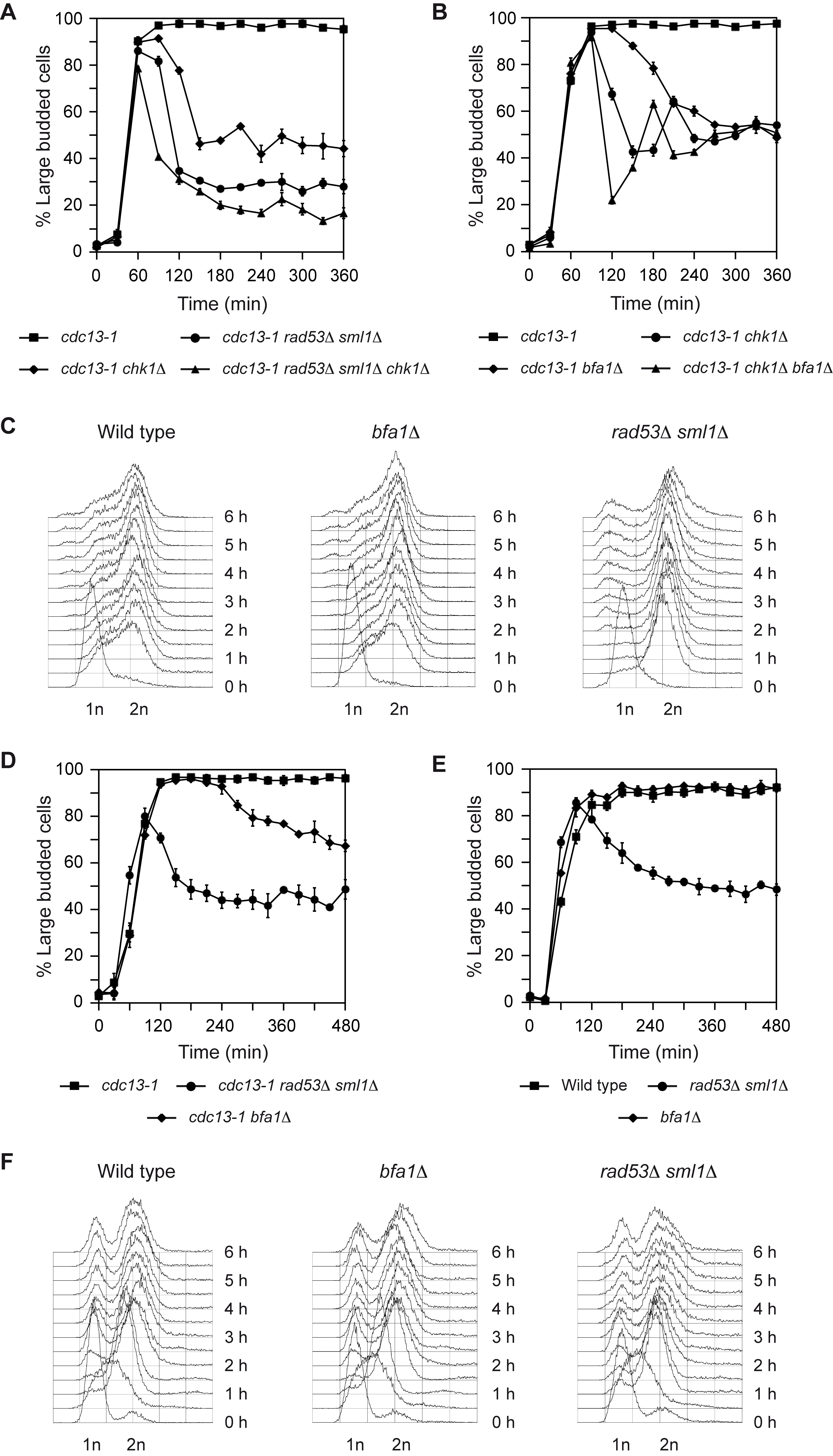

Supplement: Figure S1 — Inhibition of mitotic exit is specifically required after DNA damage to telomeres. (A–B) cdc13-1 (F965), cdc13-1 rad53Δ sml1Δ (F1662), cdc13-1 chk1Δ (F1238), cdc13-1 rad53Δ sml1Δ chk1Δ (F1830), cdc13-1 bfa1Δ (F1023), and cdc13-1 chk1Δ bfa1Δ (F1240) cells were grown in YPD at 23°C and arrested in G1 with pheromone. Cells were then released into YPD at 34°C and the percentages of large budded cells were determined at the indicated time points. Error bars indicate SD (n = 3).(C) FACS analysis corresponding to the experiment shown in Figure 1C. (D) cdc13-1 (F965), cdc13-1 rad53Δ sml1Δ (F1662) and cdc13-1 bfa1Δ (F1023) cells were grown in YPD at 23°C and arrested in G1 with pheromone. Cells were then released into YPD at 37°C and the percentages of large budded cells were determined at the indicated time points. Error bars indicate SD (n = 3). (E) Wild type (F1587), rad53Δ sml1Δ (F1019) and bfa1Δ (F1589) cells were grown in YPD at 25°C and arrested in G1 with pheromone. Cells were then released into YPD containing zeocin (50 µg/µl) at 37°C and the percentages of large budded cells were determined at the indicated time points. Error bars indicate SD (n = 3). (F) FACS analysis corresponding to the experiment shown in Figure 1D. (TIF) [file pgen.1003859.s001.tif]

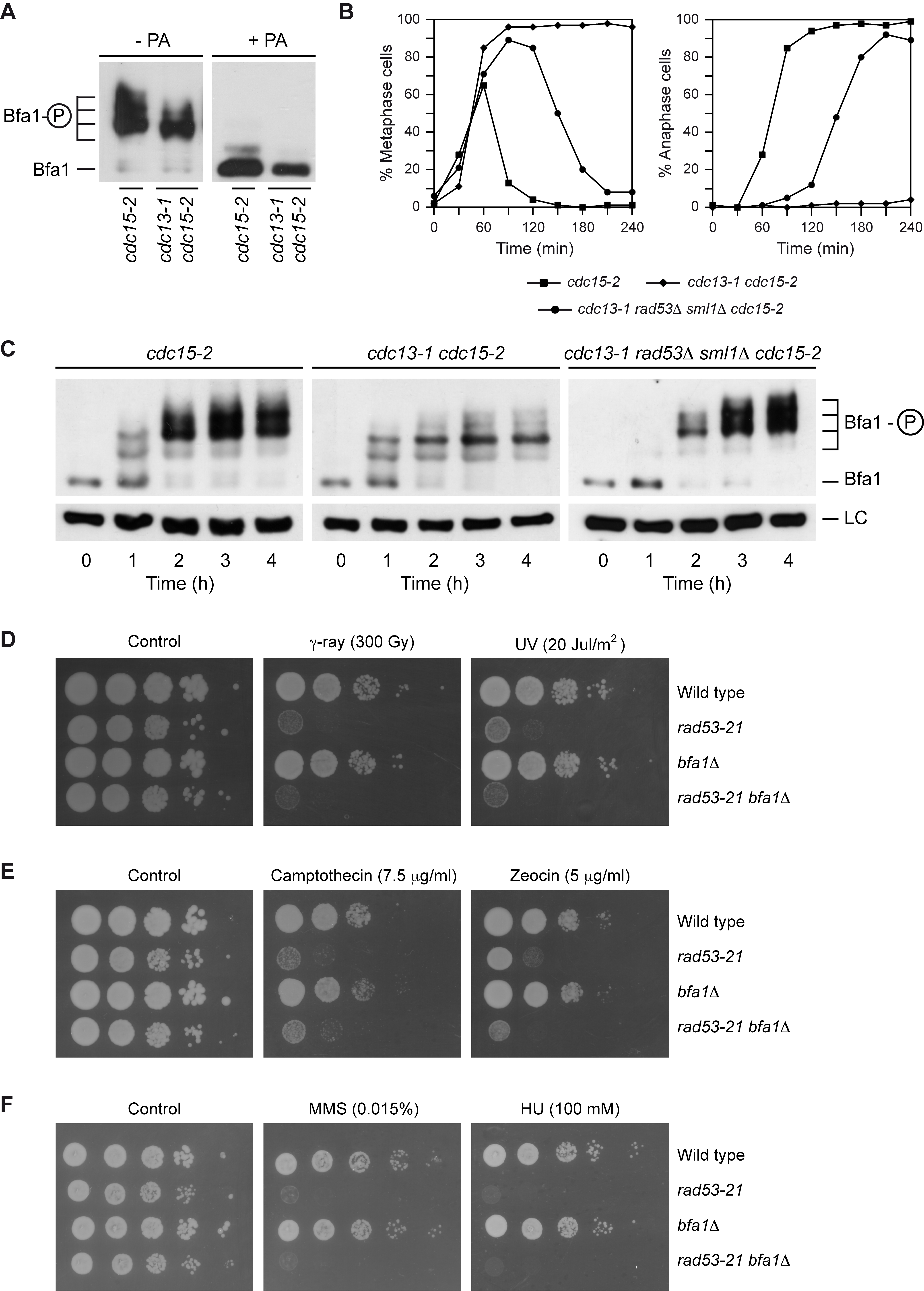

Supplement: Figure S2 — Bfa1 hypo-phosphorylation after telomere damage depends on Rad53. (A) cdc15-2 (F1492) and cdc13-1 cdc15-2 (F1488) cells expressing 3HA-Bfa1 were arrested in G1 with pheromone in YPD at 23°C and released into pheromone-free medium at 34°C for 4 h. Protein extracts were treated (+PA) or not (−PA) with phosphatase and 3HA-Bfa1 phosphorylation was determined by Western blot. (B–C) cdc15-2 (F1492), cdc13-1 cdc15-2 (F1488) and cdc13-1 rad53Δ sml1Δ cdc15-2 (F1684) cells expressing 3HA-Bfa1 were arrested in G1 with pheromone in YPD at 23°C, and then released into pheromone-free medium at 34°C. (B) The percentages of metaphase and anaphase cells were determined for each of the strains at the indicated time points. (C) 3HA-Bfa1 phosphorylation was analyzed by Western blot at the indicated time points. An unspecific band was used as a loading control (LC). (D–F) Wild type (F1587), rad53-21 (F1591), bfa1Δ (F1589) and rad53-21 bfa1Δ (F1593) cells were plated by spotting 10-fold serial dilutions of a liquid culture (OD600 = 0.3) on YPD (D–E) or minimal media (F) plates and then incubated at 30°C. (D) Before being plated, cells were irradiated with γ-rays (300 Gy) or UV (20 Jul/m2). (E–F) The cells were plated in media containing camptothecin (7.5 µg/µl), zeocin (5 µg/µl), MMS (0.015%) or HU (100 mM), as indicated. (TIF) [file pgen.1003859.s002.tif]

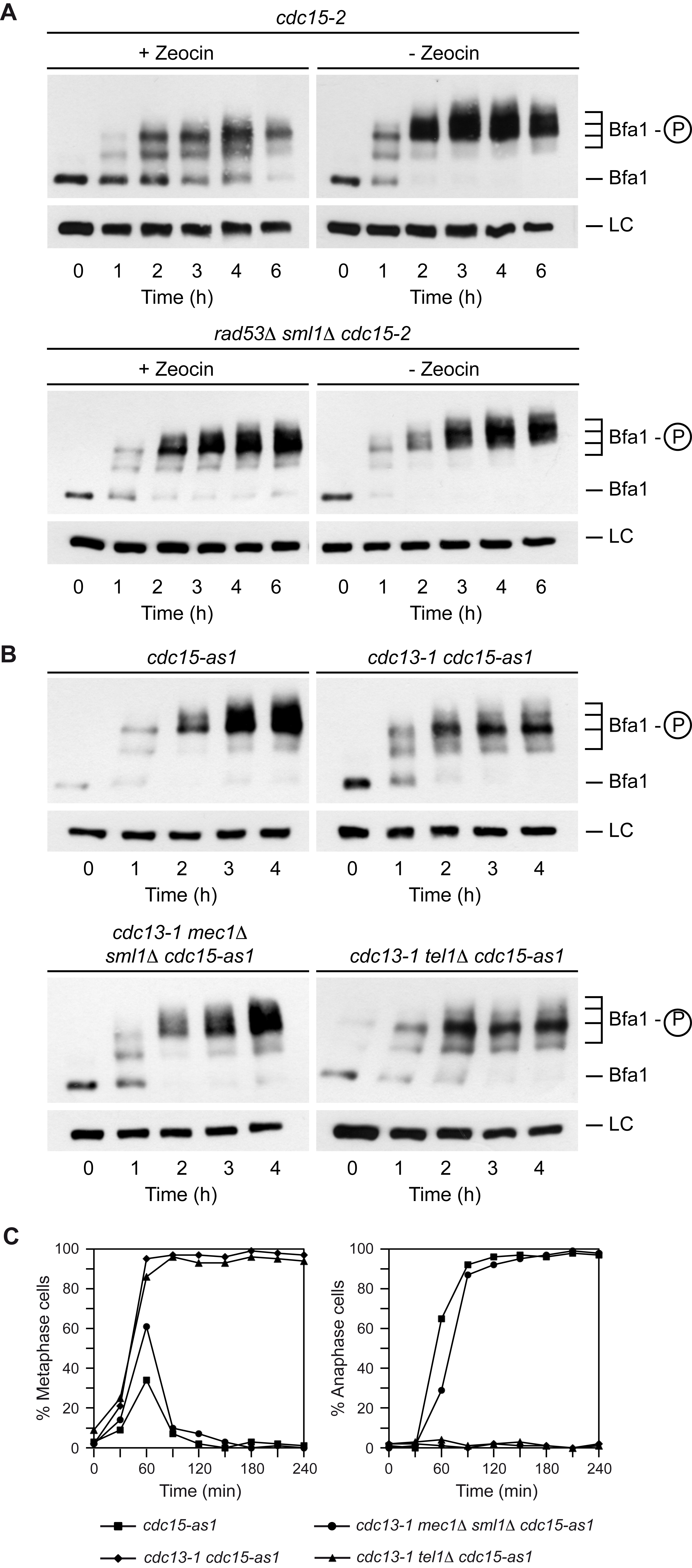

Supplement: Figure S3 — Inhibition of the hyper-phosphorylation of Bfa1 by the DDC. (A) cdc15-2 (F1492) and rad53Δ sml1Δ cdc15-2 (F1816) cells expressing 3HA-Bfa1 were arrested in G1 with pheromone in YPD at 23°C and released into pheromone-free medium at 34°C containing (+Zeocin) or not (−Zeocin) the DSB-generating compound zeocin (50 µg/ml). 3HA-Bfa1 phosphorylation was analyzed by Western blot at the indicated time points. An unspecific band was used as a loading control (LC). (B–C) cdc15-as1 (F1068), cdc13-1 cdc15-as1 (F1099), cdc13-1 mec1Δ sml1Δ cdc15-as1 (F1620) and cdc13-1 tel1Δ cdc15-as1 (F1619) cells expressing 3HA-Bfa1 were arrested in G1 with pheromone in YPD at 23°C, and then released at 34°C into pheromone-free medium containing the 1-NA-PP1 inhibitor (10 µM). (B) 3HA-Bfa1 phosphorylation was analyzed by Western blot at the indicated time points. An unspecific band was used as a loading control (LC). (C) The percentages of metaphase and anaphase cells were determined for each of the strains at the indicated time points. (TIF) [file pgen.1003859.s003.tif]

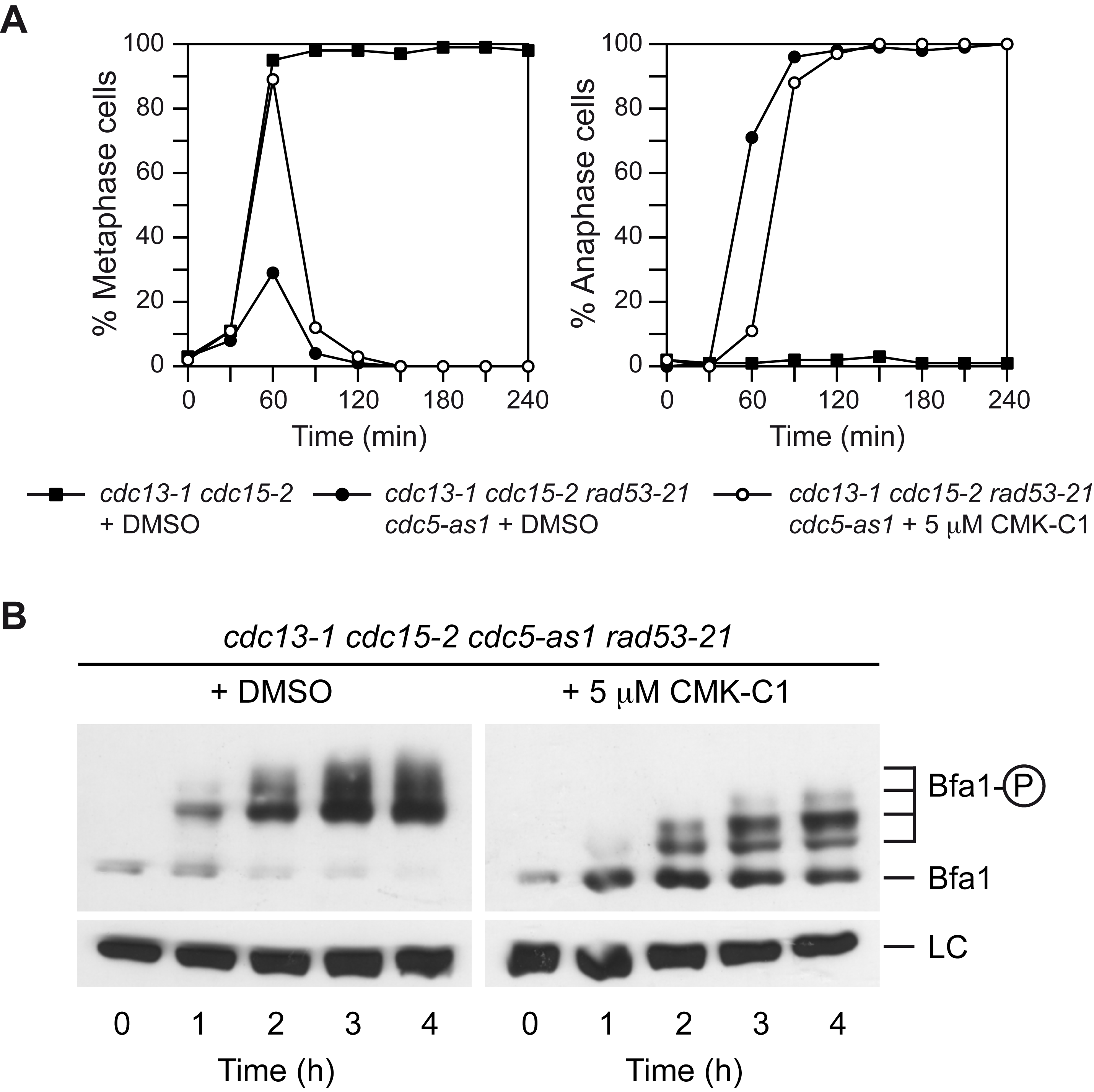

Supplement: Figure S4 — Rad53 prevents the phosphorylation of Bfa1 by Cdc5. (A–B) cdc13-1 cdc15-2 (F1488) and cdc13-1 rad53-21 cdc5-as1 cdc15-2 (F1454) cells expressing 3HA-Bfa1 were arrested in G1 with pheromone in YPD at 23°C, released into pheromone-free medium containing or not (+DMSO) the CMK-C1 inhibitor (5 µM), and incubated at 34°C. (A) The percentages of metaphase and anaphase cells were determined for each of the strains at the indicated time points. (B) 3HA-Bfa1 phosphorylation was analyzed by Western blot at the indicated time points. An unspecific band was used as a loading control (LC). (TIF) [file pgen.1003859.s004.tif]

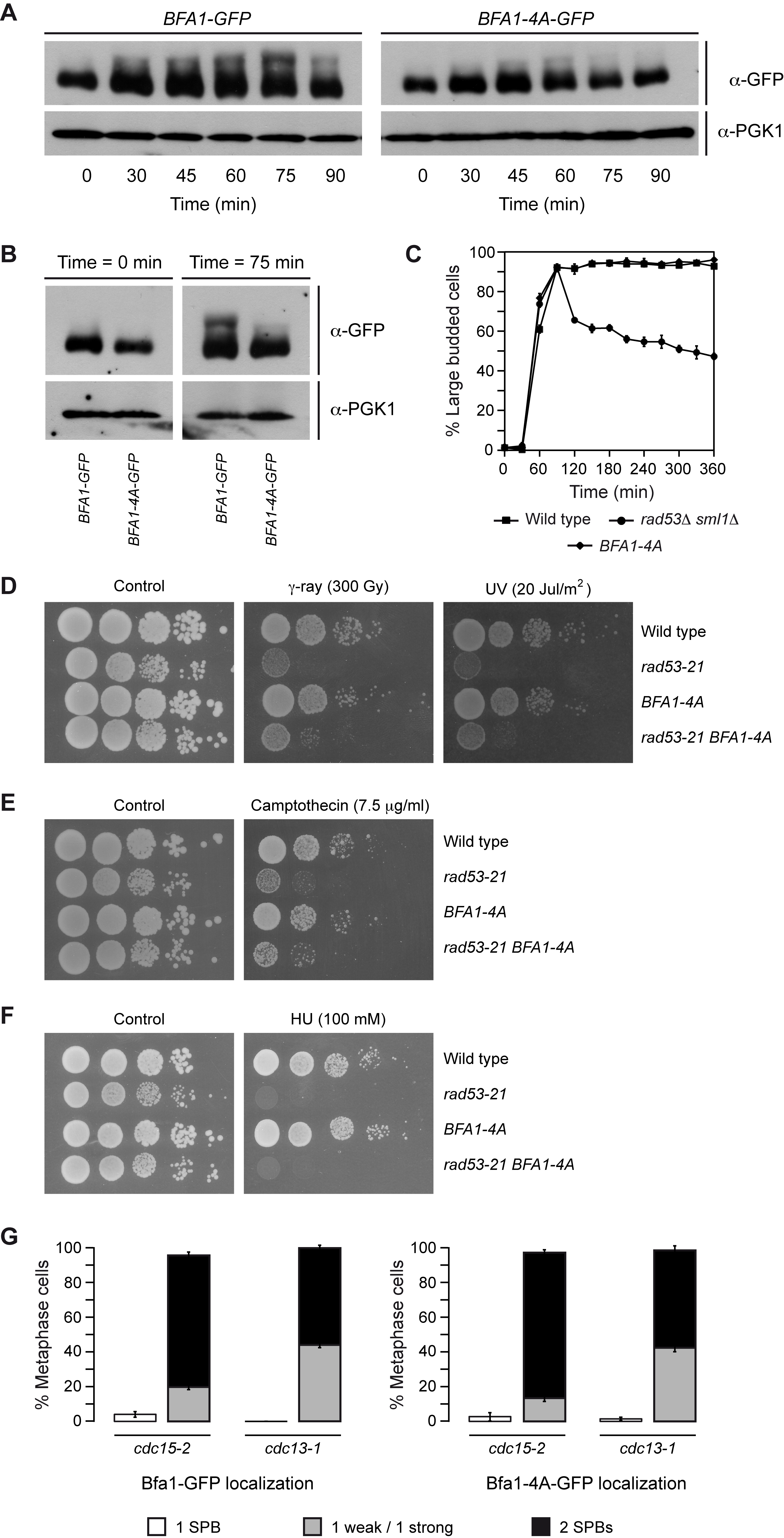

Supplement: Figure S5 — Bfa1-4A is not hyper-phosphorylated by Cdc5 and is checkpoint-proficient. (A–B) Cells expressing Bfa1-GFP (F1333) or Bfa1-4A-GFP (F1367) were arrested in G1 with pheromone in YPD at 25°C, and released into pheromone-free medium at the same temperature. Phosphorylation was analyzed by Western blot at the indicated time points with an antibody that recognizes the GFP tag (α-GFP). Pgk1 was used as a loading control (α-PGK1). (C) Wild type (F1587), rad53Δ sml1Δ (F1019) and cells expressing BFA1-4A (F1367) were grown in YPD at 25°C and arrested in G1 with pheromone. Cells were then released at the same temperature into YPD containing zeocin (50 µg/µl), and the percentages of large budded cells were determined at the indicated time points. Error bars indicate SD (n = 3). (D–F) Wild type (F1587), rad53-21 (F1591), BFA1-4A (F1367) and rad53-21 BFA1-4A (F1827) cells were plated by spotting 10-fold serial dilutions of a liquid culture (OD600 = 0.3) on YPD (D–E) or minimal media (F) plates and then incubated at 30°C. (D) Before being plated, cells were irradiated with γ-rays (300 Gy) or UV (20 Jul/m2). (E–F) The cells were plated in media containing camptothecin (7.5 µg/µl) or HU (100 mM), as indicated. (G) Analysis of Bfa1-GFP and Bfa1-4A-GFP localization to the SPBs in metaphase cells from strains F1902, F1903, F1826 and F1396 after release in YPD at 34°C from a previous G1-arrest. (TIF) [file pgen.1003859.s005.tif]
