# Supplementary material for: Inhibition of the Mitotic Exit Network in Response to Damaged Telomeres
Source: PLoS Genet. 2013 Oct 10;9(10):e1003859. doi: 10.1371/journal.pgen.1003859 (PMC3794921; doi:10.1371/journal.pgen.1003859)
Supplement: Table S1 — Strains. All strains used in this study are derivatives of W303. Unless otherwise indicated, all the strains are RAD5. (PDF) [file pgen.1003859.s006.pdf]

Supplemental Tables:

Table S1: Strains.

Strain	Relevant genotype
F965	MATa *cdc13-1*
F1662	MATa *cdc13-1 rad53::LEU2 sml1::URA3*
F1023	MATa *cdc13-1 bfa1::HIS3*
F1787	MATa *cdc13-1 bub2::HIS3*
F1786	MATa *cdc13-1 bub2::HIS3 bfa1::HIS3*
F1587	MATa wild type (this strain is W303 *RAD5*)
F1589	MATa *bfa1::HIS3*
F1019	MATa *rad53::LEU2 sml1::URA3*
F1496	MATa-inc *ade3::pGAL-HO GAL1::HOcs::hyg*
F1679	MATa-inc *ade3::pGAL-HO GAL1::HOcs::hyg rad53::LEU2 sml1::URA3*
F1606	MATa-inc *ade3::pGAL-HO GAL1::HOcs::hyg bfa1::HIS3*
F1661	MATa *rad53::LEU2 sml1::URA3 bfa1::HIS3*
F1238	MATa *cdc13-1 chk1::natR*
F1830	MATa *cdc13-1 chk1::natR rad53::LEU2 sml1::URA3*
F1240	MATa *cdc13-1 chk1::natR bfa1::HIS3*
F1492	MATa *cdc15-2 3HA-BFA1*
F1488	MATa *cdc13-1::TRP1 cdc15-2 3HA-BFA1*
F1453	MATa *cdc13-1::TRP1 cdc15-2 rad53-21 3HA-BFA1*
F1782	MATa *cdc13-1::TRP1 chk1::natR cdc15-2 3HA-BFA1*
F1784	MATa *cdc13-1::TRP1 chk1::natR rad53-21 cdc15-2 3HA-BFA1*
F1684	MATa *cdc13-1::TRP1 cdc15-2 rad53::LEU2 sml1::URA3 3HA-BFA1*
F1591	MATa *rad53-21*
F1593	MATa *rad53-21 bfa1::HIS3*
F1316	MATa *cdc13-1 rad53-21 cdc5-2::URA3 3HA-BFA1 rad5-535*
F1822	MATa *cdc15-2 3HA-CDC5*
F1824	MATa *cdc13-1::TRP1 cdc15-2 3HA-CDC5*
F1825	MATa *cdc13-1::TRP1 rad53-21 cdc15-2 3HA-CDC5*
F1816	MATa *rad53Δ sml1Δ cdc15-2 3HA-BFA1*
F1068	MATa *cdc15-as1::URA3 3HA-BFA1*

F1099	MATa *cdc13-1::TRP1 cdc15-as1::URA3 3HA-BFA1*
F1619	MATa *cdc13-1::TRP1 tel1::hyg cdc15-as1::URA3 3HA-BFA1*
F1620	MATa *cdc13-1::TRP1 mec1::LEU2 sml1::URA3 cdc15-as1::URA3 3HA-BFA1*
F1182	MATa *cdc13-1::TRP1 3HA-BFA1*
F1184	MATa *cdc13-1::TRP1 cdc28-as1::KanMX6 3HA-BFA1 rad5-535*
F1280	MATa *cdc13-1::TRP1 cdc5-L158G::KanMX6 3HA-BFA1*
F1516	MATa *cdc13-1::TRP1 cdc28-as1::HISMX6 cdc5-L158G::KanMX6 3HA-BFA1*
F973	MATa *cdc13-1 kin4::KanMX6*
F1117	MATa *cdc13-1 kin4::KanMX6 cdc15-as1::URA3 3HA-BFA1*
F1879	MATa *cdc13-1::TRP1 bub2::HIS3 cdc15-2 3HA-BFA1*
F1880	MATa *cdc13-1::TRP1 bub2::HIS3 cdc15-2 rad53-21 3HA-BFA1*
F1454	MATa *cdc13-1::TRP1 rad53-21 cdc5-L158G::KanMX6 cdc15-2 3HA-BFA1*
F1228	MATa *cdc13-1 rad53-21*
F1229	MATa *cdc13-1 rad53-21 bfa1::HIS3*
F533	MATa *bfa1::HIS3 rad5-535*
F1333	MATa *BFA1-GFP::TRP1::bfa1::HIS3 rad5-535*
F1367	MATa *BFA1-4A-GFP::TRP1::bfa1::HIS3 rad5-535*
F1315	MATa *cdc13-1 rad53-21 cdc5-2::URA3*
F1378	MATa *cdc13-1 rad53-21 cdc5-2::URA3 bfa1::HIS3*
F1396	MATa *cdc13-1 BFA1-4A-GFP::TRP1::bfa1::HIS3*
F1397	MATa *cdc13-1 rad53-21 BFA1-4A-GFP::TRP1::bfa1::HIS3*
F1827	MATa *rad53-21 BFA1-4A-GFP::TRP1::bfa1::HIS3*
F1902	MATa *cdc15-2 BFA1-GFP::TRP1::bfa1::HIS3*
F1903	MATa *cdc13-1::TRP1 cdc15-2 BFA1-GFP::TRP1::bfa1::HIS3*
F1826	MATa *cdc15-2 BFA1-4A-GFP::TRP1::bfa1::HIS3*
